# Extensive Wound Management of a Retroperitoneal Abscess Due to Complicated Pyelonephritis in a Patient With Type 2 Diabetes: A Case Report

**DOI:** 10.7759/cureus.94203

**Published:** 2025-10-09

**Authors:** Benjamin Jesus Moya-Leal, Luis Adrian Alvarez-Lozada, Diego Escamilla-Magaña, Hugo Enrique Hernandez-Gamboa, Maria Fernanda Balderas-Sandoval

**Affiliations:** 1 General Surgery, Institute for Social Security and Services for State Workers (ISSSTE) Monterrey Regional Hospital, Monterrey, MEX; 2 Human Anatomy, Facultad de Medicina - Universidad Autónoma de Nuevo León, Monterrey, MEX

**Keywords:** abscess, hypochlorous acid, negative-pressure wound therapy, skin graft, type 2 diabetes

## Abstract

Complicated urinary tract infections can progress to deep-seated abscesses, particularly in patients with comorbidities such as type 2 diabetes mellitus. These infections may result in severe soft tissue damage and require a complex, comprehensive and multidisciplinary approach to achieve favorable clinical outcomes.

A 42-year-old male with poorly controlled type 2 diabetes mellitus developed a retroperitoneal abscess secondary to pyelonephritis, extending to the psoas muscle with cutaneous exteriorization in the left thigh region. Upon admission, he presented with an extensive purulent wound, hypoalbuminemia, and electrolyte imbalances. Management included antibiotic therapy, nutritional support, local wound care with negative pressure therapy, and acetic acid. A split-thickness skin graft was later performed, with successful integration. The patient experienced a favorable clinical course, with progressive functional recovery and no infectious complications. This case highlights the importance of a comprehensive and staged approach in managing complex wounds resulting from deep infections in vulnerable patients. The combination of local antimicrobial treatment, negative pressure therapy, and skin grafting enabled effective recovery, without the need for extensive surgical interventions.

## Introduction

Complications of urinary tract infections in patients with diabetes mellitus are often more severe and complex than in non-diabetic individuals [1]. Reports documenting progression to extensive cutaneous wounds are exceedingly scarce. This underscores the rarity of this clinical presentation and highlights the need to disseminate experiences that may help guide diagnostic and therapeutic approaches in similar scenarios. In advanced stages, these infections can lead to extensive soft tissue involvement, including retroperitoneal abscesses that, due to their anatomical location, may dissect along fascial planes and track through the psoas muscle. In such scenarios, a multidisciplinary approach becomes essential.

Patients with type 2 diabetes mellitus frequently present with alterations across multiple physiological systems - including immunological dysfunction, vascular compromise, and impaired wound healing - which contribute to the severity and chronicity of infections [2]. Retroperitoneal abscesses represent a particularly challenging clinical entity. Their incidence has been reported to be 0.3%-0.4% in cases associated with perforated colorectal cancer [3], with a higher prevalence among patients with diabetes [4]. Given the anatomical disposition of the retroperitoneal space, spontaneous drainage may occur through the thigh, further complicating the clinical picture and necessitating a tailored and coordinated management plan [5].

The available literature on this topic is largely limited to isolated case descriptions, such as the report by van den Berge et al. (2005), which documented several instances of psoas abscesses - only two of which occurred in patients with diabetes and were not secondary to a urinary tract infection [6]. In contrast, we describe a case of a retroperitoneal abscess secondary to pyelonephritis, extending through the psoas muscle in a malnourished patient with poorly controlled type 2 diabetes mellitus. The complexity of this presentation required a comprehensive, multidisciplinary approach to achieve a favorable clinical outcome.

## Case presentation

The patient was a 42-year-old male with a seven-year history of type 2 diabetes mellitus, managed solely with insulin. Relevant medical history included active smoking (one pack per day since the age of 19) and regular alcohol consumption. Immunodeficiency testing, including HIV serology, was negative.

The clinical presentation began with left lumbar pain, accompanied by symptoms suggestive of an upper urinary tract infection. Although no imaging studies were performed at the time to confirm the diagnosis, the condition progressed to a retroperitoneal abscess that extended through the left psoas muscle, resulting in an open cutaneous lesion involving the left hip and thigh regions. Due to the complexity of the case, the patient was referred to our institution for specialized management.

Upon admission, the patient was alert and oriented but exhibited signs of significant malnutrition and premature aging. He was unable to ambulate due to his current clinical condition. Abdominal examination showed no tenderness or palpable masses. Inspection of the extremities revealed a large, bloody wound on the left thigh, with mild purulent discharge. There were no signs of necrosis or cellulitis.

Table 1 summarizes the initial laboratory tests, which demonstrated anemia and critically low glucose levels. Renal function parameters were within normal limits; however, the erythrocyte sedimentation rate (ESR) was slightly elevated. Serum electrolytes revealed fluid and electrolyte imbalances, including hyponatremia and mild hypochloremia. Additional findings included hypocalcemia, mild hyperphosphatemia, decreased procalcitonin, and elevated alkaline phosphatase. Urinalysis showed abundant bacteria, consistent with a urinary tract infection. Upon admission, a wound culture was obtained, yielding growth of Staphylococcus epidermidis along with a positive urinalysis.

**Table 1:** Initial laboratory tests BUN, blood urea mitrogen; µL, microliter; g/dL, grams per deciliter; mg/dL, milligrams per deciliter; mm/h, millimeters per hour; mEq/L, milliequivalents per liter; ng/mL, nanograms per milliliter; IU/L, International Units per liter

Laboratory Test	Result	Reference Range
White blood cell count	7.03 /µL	4.0 - 11.0 /µL
Hemoglobin	10.1 g/dL	13.5 - 17.5 g/dL
Glucose	48 mg/dL	70 - 100 mg/dL
Creatinine	0.4 mg/dL	0.6 - 1.3 mg/dL
Urea (BUN)	14 mg/dL	7 - 20 mg/dL
Erythrocyte sedimentation rate (ESR)	18 mm/h	<15 mm/h
Sodium	129 mEq/L	135 - 145 mEq/L
Potassium	4.0 mEq/L	3.5 - 5.0 mEq/L
Chloride	96 mEq/L	98 - 106 mEq/L
Calcium	6.8 mg/dL	8.5 - 10.5 mg/dL
Magnesium	1.5 mg/dL	1.5 - 2.5 mg/dL
Phosphate	4.7 mg/dL	2.5 - 4.5 mg/dL
Procalcitonin	0.056 ng/mL	<0.1 ng/mL
Alkaline phosphatase	208 IU/L	44 - 147 IU/L

**Figure 1:**
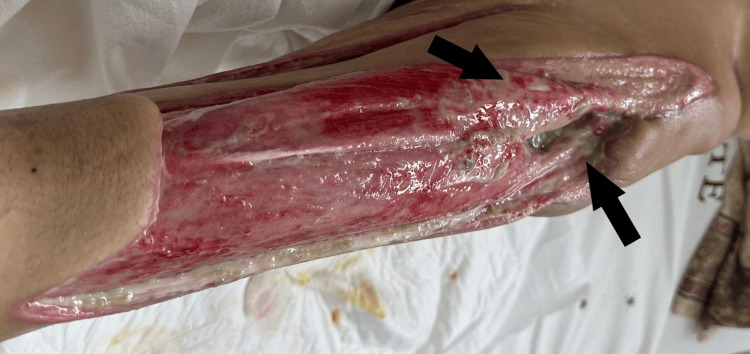
Extensive wound throughout the left thigh Initial debridement was performed for the wound (black arrows), which extended throughout the left thigh.

**Figure 2:**
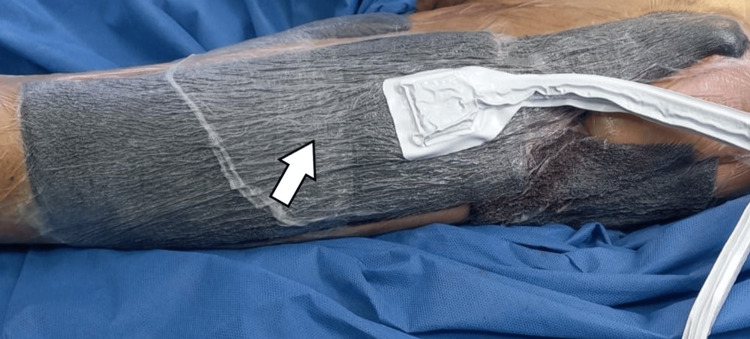
Negative pressure wound therapy A vacuum-assisted closure (VAC) system was applied for negative pressure wound therapy, with dressing changes performed every three to four days. The white arrow indicates the foam dressing of the VAC system covering the wound, which is overlaid with an adhesive drape to maintain negative pressure and facilitate fluid removal.

Upon admission to our unit, the patient received correction of electrolyte imbalances and specialized nutritional support. Given the confirmed bacterial infection, intravenous piperacillin-tazobactam (4.5 g every six hours) was initiated and continued for 10 days after admission. Local wound management initially consisted of daily dressings with gauze impregnated with Dakin’s solution (diluted sodium hypochlorite) during the first week (Figure 1). This was followed by the application of a RENASYS negative pressure wound therapy (NPWT) system (Smith & Nephew plc, London, United Kingdom), with dressing changes every three to four days for 12 days (Figure 2), after which it was discontinued as per therapeutic indication. Daily wound care with Dakin’s solution was then resumed for one week, after which negative pressure therapy was reintroduced, with dressing changes every three to four days for an additional 12 days.

**Figure 3:**
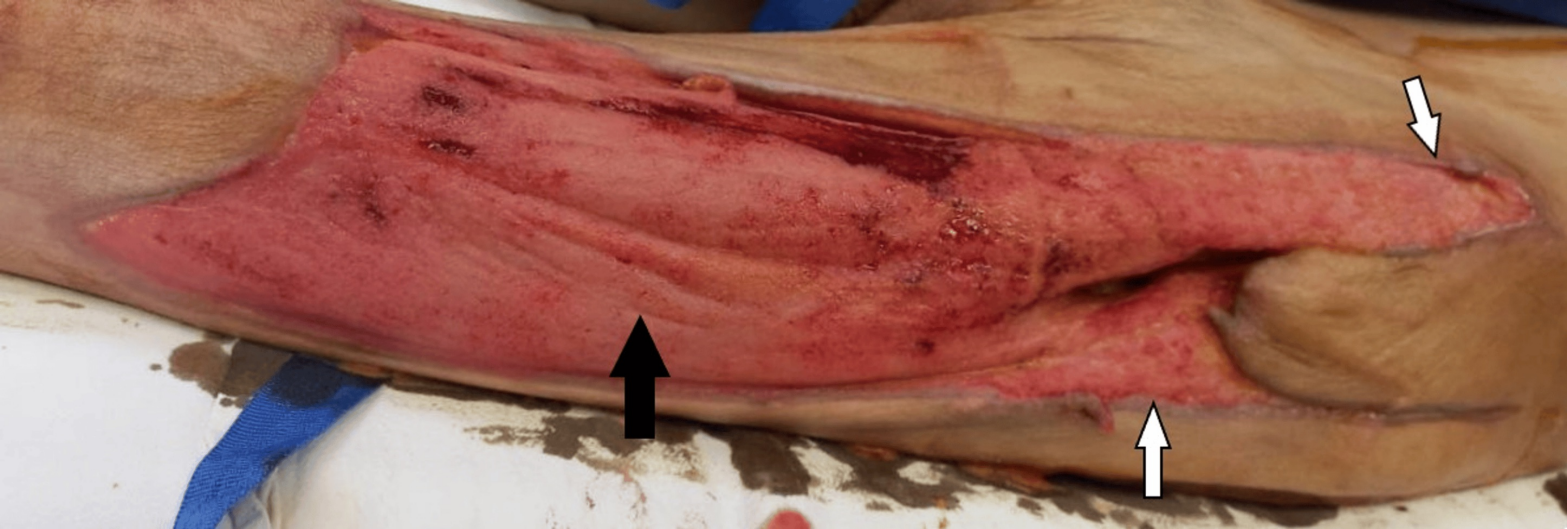
Granulation tissue Sufficient granulation tissue was obtained. The black arrow indicates healthy granulation tissue following vacuum-assisted closure (VAC) system therapy, while the white arrows indicate the undermined edges of the wound.

**Figure 4:**
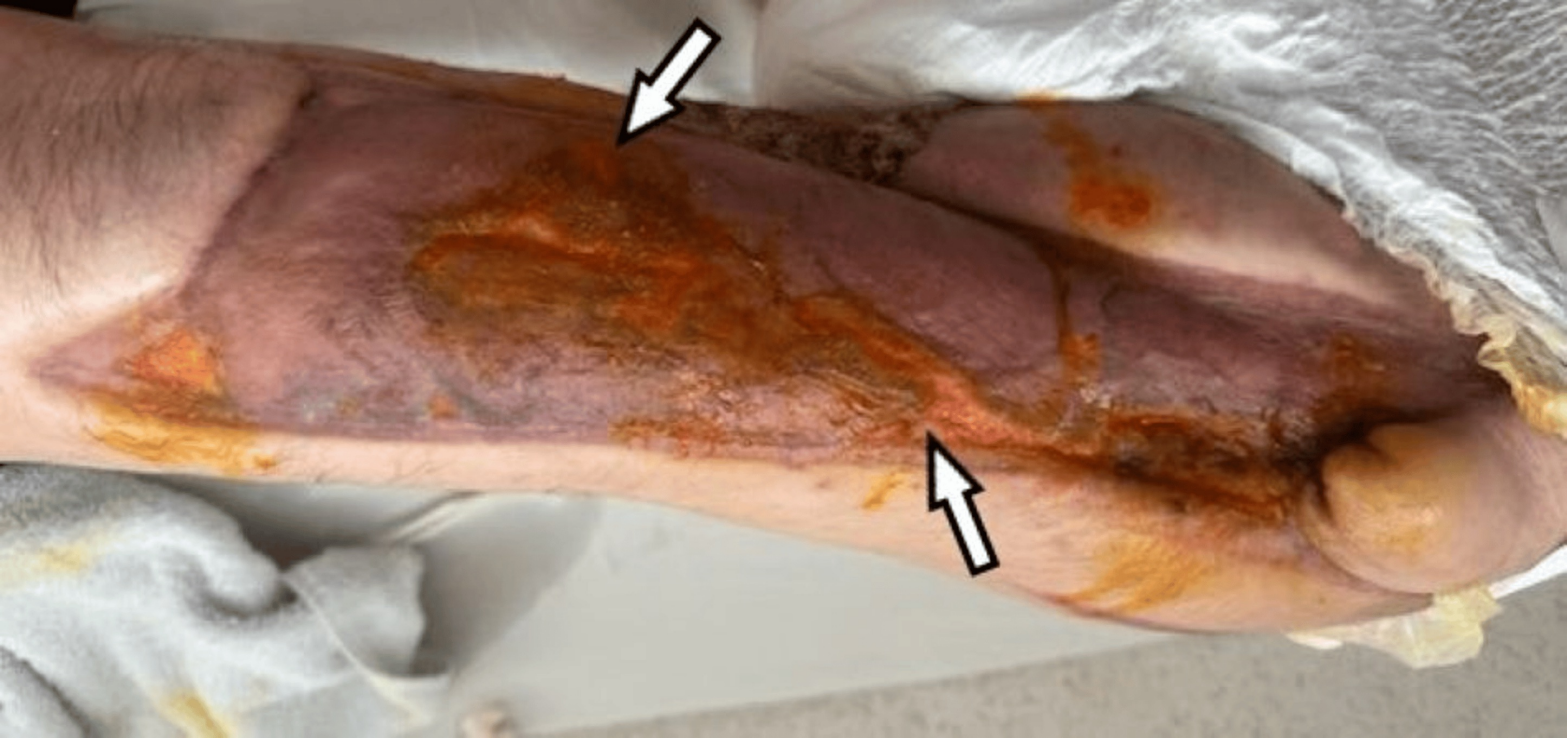
Skin graft A split-thickness skin graft (white arrows) was performed to achieve definitive wound coverage.

Following the second removal of the negative pressure system, care continued with acetic acid dressings until discharge, achieving an adequate granulation tissue base (Figure 3), after which a partial-thickness skin graft was performed using a dermatome. Hospitalization lasted two months, during which the patient demonstrated favorable progress without further infectious episodes, and was ultimately discharged due to clinical improvement (Figure 4).

## Discussion

This case describes a patient with type 2 diabetes mellitus who developed a retroperitoneal abscess secondary to complicated pyelonephritis, an infrequent clinical scenario that remains poorly documented in current medical literature. Although some similar cases have been reported [6,7], the occurrence of a retroperitoneal abscess secondary to pyelonephritis continues to be rare. Furthermore, there is a notable lack of evidence regarding the clinical and surgical management of these presentations. While imaging studies typically play a key role in diagnosis, in this case, the lesion was clinically identified following spontaneous cutaneous exteriorization.

In patients with type 2 diabetes mellitus, wound healing is impaired due to alterations in the normal repair process, which prolongs healing time. A sustained inflammatory state, impaired new blood vessel formation, and abnormal scar remodeling lead to delayed healing, increased risk of chronic wounds, pathological scarring, and infections. These factors contribute to higher rates of wound dehiscence, poor healing, and surgical site infections in diabetic patients [2].

In this case, the presence of hypoglycemia, mild anemia, and electrolyte disturbances reflects a catabolic and malnourished state, which likely contributed to delayed wound healing and an increased risk of infectious complications. The mildly elevated ESR and increased alkaline phosphatase further support the presence of chronic inflammation and heightened tissue catabolism.

The management of this case required careful consideration of the patient's underlying malnutrition, which compromises the immune response and predisposes patients to more severe and prolonged infections [8], as occurred in this case with the retroperitoneal abscess. The impairment of wound healing is typically slower and less effective due to the multiple metabolic alterations associated with both conditions [8]. Consequently, the choice of surgical interventions, such as skin grafting, represented an additional clinical challenge that required an individualized and multidisciplinary approach.

The relevance of this case lies in the therapeutic decision to employ a skin graft, a strategy that may have significant clinical implications in the management of patients with diabetes mellitus, particularly in settings of severe infection, malnutrition, or multiple comorbidities [9]. According to the literature, diabetic patients undergoing split-thickness skin grafts (STSGs) face up to a fivefold increased risk of developing postoperative complications compared to non-diabetic patients [10]. The failure rate of this type of graft ranges between 19% and 30%, with a higher incidence of poor graft integration, delayed wound healing, and the need for surgical reinterventions [11,12].

The success of the graft largely depends on meticulous preparation of the recipient bed, which includes optimal nutritional status, effective disinfection, adequate development of granulation tissue, proper vascular perfusion, as well as appropriate surgical techniques and postoperative care. Any mechanical or biological disruption of these conditions can significantly compromise graft viability [13].

This case highlights the importance of a multidisciplinary approach that incorporates the assessment and correction of nutritional status as a key component of comprehensive patient management. The coexistence of type 2 diabetes and malnutrition increases the risk of infectious complications and hinders recovery, constituting a clinical challenge that exceeds the scope of any single medical specialty [14].

Although, in this case, the wound culture yielded *Staphylococcus epidermidis* - typically regarded as a skin contaminant - in the context of an extensive open wound with a contiguous abscess, this finding may represent a true opportunistic pathogen, thereby justifying targeted antibiotic therapy. Additionally, urinary findings confirmed a urinary tract infection with retroperitoneal extension to the left psoas muscle.

International guidelines support that multidisciplinary teams composed of specialists in surgery, infectious diseases, clinical nutrition, endocrinology, and advanced wound care in diabetic patients contribute significantly to the reduction of healing time, length of hospital stay, amputation rates, and the incidence of infectious complications [14]. In conjunction with multidisciplinary management, the use of skin grafts promotes earlier wound healing, with low failure rates, reduced recurrence, and fewer complications [15].

In diabetic patients, the use of diluted Dakin’s solution (0.1%-0.5%) in infected wounds has been described in the literature as an effective tool for infection control and granulation tissue promotion, without significantly impairing the healing process [16-18]. Dakin’s solution was initially used as an antiseptic agent to reduce microbial load while preserving viable tissue. This was followed by NPWT (vacuum-assisted closure, or VAC), which promoted granulation tissue formation, prevented bacterial colonization, and enhanced the effectiveness of systemic antibiotic therapy [19,20]. Finally, the skin graft provided effective coverage of the defect.

The NPWT intervention promoted progressive granulation tissue formation, achieving complete wound coverage in approximately five weeks. Integration of the STSG reached 90%, demonstrating the effectiveness of the multidisciplinary and staged approach implemented in this case.

This staged approach yielded satisfactory clinical outcomes and prevented delays in wound healing, while also avoiding the need for extensive surgical interventions. The case illustrates the importance of a multidisciplinary approach, close clinical monitoring, and comprehensive management - including metabolic and nutritional rehabilitation - as essential elements for recovery in vulnerable patients [15].

## Conclusions

Complicated urinary tract infections may progress to severe presentations, including deep abscesses with cutaneous involvement. An integrated management approach that combines advanced local wound care, systemic antibiotic therapy, and metabolic support is essential for optimal outcomes. Dakin’s solution serves as a valuable tool in controlling the initial microbial load in complex wounds. Furthermore, NPWT, along with skin grafting, facilitates accelerated healing and enhances functional prognosis.

## References

[1] Brook I (2008). Microbiology and management of soft tissue and muscle infections. Int J Surg.

[2] Dasari N, Jiang A, Skochdopole A, Chung J, Reece EM, Vorstenbosch J, Winocour S (2021). Updates in diabetic wound healing, inflammation, and scarring. Semin Plast Surg.

[3] Zhou J, Wan S, Li C, Ding Z, Qian Q, Yu H, Li D (2023). Retroperitoneal abscess as a presentation of colon cancer: the largest case set analysis to date, which extracted from our unit and the literature. Front Oncol.

[4] Mallia AJ, Ashwood N, Arealis G, Galanopoulos I (2015). Retroperitoneal abscess: an extra-abdominal manifestation. BMJ Case Rep.

[5] Zhou Z, Song Y, Cai Q, Zeng J (2010). Primary psoas abscess extending to thigh adductors: case report. BMC Musculoskelet Disord.

[6] van den Berge M, de Marie S, Kuipers T, Jansz AR, Bravenboer B (2005). Psoas abscess: report of a series and review of the literature. Neth J Med.

[7] Kamikado C, Taguchi S, Wakiyama T (2009). Psoas abscess and bacterial peritonitis caused by urinary tract infection in a patient of liver cirrhosis and diabetes mellitus. Clin J Gastroenterol.

[8] Zhang SS, Tang ZY, Fang P, Qian HJ, Xu L, Ning G (2013). Nutritional status deteriorates as the severity of diabetic foot ulcers increases and independently associates with prognosis. Exp Ther Med.

[9] Dias RH, Salelkar R, Rodrigues J, Rodrigues FCS, Parsekar S (2023). A clinicopathological study on split thickness skin graft uptake in diabetics and factors affecting graft uptake. World J Surg Surgical Res.

[10] Donegan RJ, Schmidt BM, Blume PA (2014). An overview of factors maximizing successful split-thickness skin grafting in diabetic wounds. Diabet Foot Ankle.

[11] Lamani YP, Reddy MA, Kalburgi EB, Suhas BS (2020). Comparison of split skin thickness graft survival in diabetic and non-diabetic ulcer. Int Surg J.

[12] Reddy S, El-Haddawi F, Fancourt M (2014). The incidence and risk factors for lower limb skin graft failure. Dermatol Res Pract.

[13] Ayaz M, Ayaz D, Kheshti A (2025). Burn wounds on diabetic feet: early excision and grafting vs. conventional care in preventing diabetic foot progression - a cluster-randomized trial. Discov Med.

[14] (2025). IWGDF guidelines on the prevention and management of diabetic foot disease. https://iwgdfguidelines.org/wp-content/uploads/2019/05/IWGDF-Guidelines-2019.pdf.

[15] Yammine K, Assi C (2019). A meta-analysis of the outcomes of split-thickness skin graft on diabetic leg and foot ulcers. Int J Low Extrem Wounds.

[16] Armstrong DG, Lavery LA (2025). Diabetic foot infections: treatment & management. Medscape.

[17] Duarte B, Formiga A, Neves J (2020). Dakin's solution in the treatment of severe diabetic foot infections. Int Wound J.

[18] Ramírez Solís ME, Cárdenas Lailson LLE, Torres GB, Domínguez JGL, Athié Athié AAJ, Mijares GJM (2000). Comparative study of the usefulness of acetic acid vs. modified Dakin's solution in incisional site infections (Article in Spanish). Cir Gen.

[19] Jiang ZY, Yu XT, Liao XC (2021). Negative-pressure wound therapy in skin grafts: a systematic review and meta-analysis of randomized controlled trials. Burns.

[20] Biermann N, Wallner S, Martini T, Spoerl S, Prantl L, Taeger CD (2023). Negative pressure wound therapy: analysis of the rinsing fluid as a monitoring tool and approach to the inflammatory process: a pilot study. J Clin Med.

